# IL-23 and Th17 Disease in Inflammatory Arthritis

**DOI:** 10.3390/jcm6090081

**Published:** 2017-08-29

**Authors:** Toru Yago, Yuki Nanke, Manabu Kawamoto, Tsuyoshi Kobashigawa, Hisashi Yamanaka, Shigeru Kotake

**Affiliations:** Institute of Rheumatology, Tokyo Women’s Medical University 10-22 Kawada-cho, Shinjuku-ku, Tokyo 162-0054, Japan; ynn@twmu.ac.jp (Y.N.); kawamoto@twmu.ac.jp (M.K.); tkobashigawa@twmu.ac.jp (T.K.); yamanaka@twmu.ac.jp (H.Y.); skotake@twmu.ac.jp (S.K.)

**Keywords:** IL-23, rheumatoid arthritis, spondyloarthritis, ankylosing spondylitis, psoriatic arthritis

## Abstract

IL-23, which is composed of p19 and p40 subunits, is a proinflammatory cytokine that contributes to the formation and maintenance of Th17 cells in inflammatory autoimmune diseases. IL-23 is a human osteoclastogenic cytokine and anti-IL-23 antibody attenuates paw volume and joint destruction in CIA rats. IL-23 levels in serum and synovial fluid are high in rheumatoid arthritis (RA) patients, and IL-23 may be a useful biomarker for the diagnosis of RA. In addition, IL-23 affects the pathogenesis of inflammation and bone destruction through interaction with other cytokines such as IL-17 and TNF-α. Furthermore, polymorphisms of *IL23R* are a risk factor for ankylosing spondylitis (AS) and psoriatic arthritis (PsA), which indicates that IL-23 is also involved in the pathogenesis of spondyloarthritis (SpA). Finally, IL-17 and IL-23 inhibitors reduce the clinical manifestations of SpA. Thus, the IL-23/Th17 pathway is a therapeutic target for the treatment of inflammatory arthritis.

## 1. IL-23 and Th17 cells

IL-23 was identified in 2000 as a heterodimeric, proinflammatory cytokine and a member of the IL-12 family [1]. IL-23 is mainly secreted by antigen-presenting cells, is composed of the p19 and p40 subunits, and shares the p40 subunit with IL-12 [1]. IL-23 signals are transferred through the IL-23 receptor complex, which is composed of the IL-23 receptor and the IL-12 receptor β chain [2]. IL-23 induces the expression of IFN-γ in human CD45RO-positive (memory) T cells and activated memory T cells to secrete several inflammatory cytokines including IL-17 and IFN-γ [1,3]. Recombinant human IL-23 increases the production of IFN-γ, IL-10, and IL-17 in activated human naïve T cells [4]. In models of human T helper type 1 (Th1) differentiation, IL-23 acts after IL-12 and maintains Th1 commitment by its preferential action on memory T cells [5,6,7]. In animal studies, Cua et al. reported that IL-23-deficient (IL-23p19^−/−^) mice are resistant to experimental autoimmune encephalomyelitis [8]. However, IL-12 (p35)-deficient mice are still susceptible to inflammation [8]. In addition, Murphy and colleagues reported that mice with collagen-induced arthritis (CIA) and IL-23 deficiency (IL-23 p19^−/−^) are completely resistant to the development of joint destruction and that IL-23 is required for the induction of inflammatory cytokines including IL-17 and TNF-α [9]. Moreover, transgenic mice constitutively overexpressing IL-23 p19 develop spontaneous severe multi-organ inflammation with increased levels of TNF-α [10]. These findings suggest that the IL-23/IL-17 pathway has an essential role in the establishment and maintenance of inflammatory autoimmune diseases and emphasize the importance of understanding the origins of developing IL-17 effector cells [3,11]. 

In 2005, Th17 cells were reported to be a novel subset of effector Th cells [12,13], which are induced by IL-23 stimulation with anti-IFN-γ and anti-IL-4 antibodies. In human immune cells, IL-23 receptors are expressed on activated or memory T cells, on natural killer cells, and, to a lesser extent, on dendritic cells and macrophages [2]. In addition to the expression of specific patterns of chemokine receptors, such as C-C chemokine receptor type 6 (CCR6) and CCR4 [14], several reports demonstrated that IL-23R is a specific marker for the Th17 population [15,16]. It is thought that IL-23 is not required for early Th17 development because IL-23 receptor is not expressed on naïve Th cells. Bettelli et al. reported that IL-23 is not a differentiation factor for the generation of mouse Th17 cells [17]. Importantly, IL-23 is necessary for the maintenance and pathogenicity of Th17 cells [18]. Moreover, O’Shea and colleagues found that IL-23 markedly upregulates the expression of IL-23R on “memory” helper T cells and is an important inducer for IL-17 production [19]. In addition, Sato et al. observed that Th17 cells can become osteoclastogenic T helper cell subtypes and that IL-23 induces the expression of IL-17 and receptor activator of NF-κB ligand (RANKL) on CD4 T cells in mice [20]. Recently, Blimp-1, which is induced by IL-23, was reported to promote the pathogenicity of Th17 cells [21]. 

## 2. IL-23 and Inflammatory Arthritis

### 2.1. IL-23 and Rheumatoid Arthritis

IL-23 is thought to promote joint swelling and destruction in RA patients by two processes: (1) inflammation and (2) bone destruction. The mechanism of these two effects is caused by IL-23-induced stimulation of IL-17. Many studies have provided evidence that IL-17 appears to be involved in the development and maintenance of chronic inflammatory diseases [22]. It should be noted that we call “IL-17A” “IL-17” in the current review. IL-17 increases the expression and production of TNF-α and IL-1β by human macrophages [23], and induces production of IL-1β in osteoblasts [24]. In 1999, we have already reported that IL-17 levels in synovial fluids are significantly higher in RA patients than in osteoarthritis patients and that IL-17 induces murine osteoclastogenesis by inducing the expression of RANKL via a mechanism involving the synthesis of prostaglandin E2 in osteoblasts in vitro [25]. In addition, IL-17 directly induces human osteoclast differentiation from human monocytes alone, via the TNF-α or the RANK–RANKL pathway in 2009 [26].

Raza et al. found that early RA patients whose disease duration was less than three months (mean nine weeks) have a clear and transient cytokine profile of T cells in synovial fluid, including IL-17, but not IFN-γ in 2005 [27]. This study indicates that disease duration is important for the role of cytokines in the pathogenesis of inflammatory diseases, such as RA, and suggest that regulation of IL-23 may prevent human osteoclastogenesis or joint inflammation in RA. 

We therefore hypothesized that IL-23 induces human osteoclastogenesis and that anti-IL-23 antibody inhibits joint inflammation. We demonstrated that IL-23 directly induces human osteoclast differentiation in cultures of PBMC in the absence of exogenous sRANKL or osteoblasts [28]. To elucidate the factors involved in IL-23-induced osteoclast differentiation from PBMC, we used various inhibitors including osteoprotegerin (a decoy receptor for RANKL), anti-IL-17 antibody, and etanercept (a TNF-α inhibitor). All inhibitors blocked IL-23-induced osteoclast differentiation, even at 1.0 ng/ml and it was the most effective concentration of IL-23 for inducing osteoclast differentiation. These results suggest that several cytokines including RANKL, IL-17, and TNF-α are, at least partly, involved with bone destruction of RA by IL-23-induced osteoclast differentiation. 

Moreover, we demonstrated that IL-17 production, but not IFN-γ, is dose-dependently induced from human activated T cells stimulated with IL-23 [28]. The balance between the inducible effect of IL-17 [26] and the inhibitory effect of IFN-γ [29] may be critical in IL-23-induced osteoclast differentiation. Our findings also indicate that IL-23 administered at a later stage significantly reduces paw volume dose-dependently in CIA rats. Moreover, anti-IL-23 antibody also reduces synovial tissue inflammation and bone destruction in CIA rats. Therefore, our report is the first to demonstrate that IL-23 could be a therapeutic target and that IL-23 inhibitor has the potential to attenuate synovial inflammation and joint destruction even after onset of RA [28]. Contradictory results showed that anti-IL-23 antibody does not have a significant curative effect in CIA mice [30]; however, our experiments clearly demonstrated that anti-IL-23 antibody administration significantly decreases paw volume in CIA rats and prevents joint destruction by X-ray evaluation. The reason for these discrepancies is unclear. One possibility is the difference in antibodies used in each experiment. 

There are some studies that support the role of IL-23 in RA patients. In 2007, Kim et al. found that IL-23p19 levels in sera and synovial fluid are higher in patients with RA than in OA patients or healthy controls [31]. They also showed that IL-17 stimulates IL-23p19 mRNA and protein expression in synovial fibroblasts with RA. Moreover, Liu et al. reported that TNF-α and IL-1β stimulated the production of IL-23p19 from RA fibroblast-like synoviocytes (FLS) [32], and Goldberg et al. reported that TNF-α and IL-17 synergistically induce IL-23p19 mRNA expression in FLS [33]. These studies strongly suggest that there is a malignant cycle with various proinflammatory cytokines, such as IL-23, IL-17, IL-1β, and TNF-α in the RA synovium. Wendling et al. found a positive correlation between serum IL-23 and IL-6 in patients with RA in 2015 [34]. Interestingly, they showed that serum IL-23 levels positively correlate with cytoplasmic Sirt1 activity and that increased apoptosis rate in PBMC with RA negatively correlates with the expression of serum IL-23 levels and Sirt1 protein. They concluded that dysregulation of serum IL-23 and Sirt1 expression and activity in RA parallels increased PBMC apoptosis. 

Recently, Zaky et al. reported that IL-23 levels in serum are significantly elevated in RA patients compared with healthy controls [35]. They have suggested that IL-23 levels are not correlated to 28-joint disease activity scores, which indicates that IL-23 could be a useful biomarker for diagnostic purposes of RA. Furthermore, it has been shown that IL-23p19 concentration correlates with that of IL-17 in synovial fluid and sera, and with that of TNF-α and IL-1β in the sera of 22 patients with RA [36]. Interestingly, the same group showed that the concentration of IL-23p19 measured by ELISA in synovial fluid is higher in RA patients who had bone erosions than in those who had no erosions [36]. 

From this point of view, IL-23 inhibitors were tried for treatment of RA, but use of guselkumab, a human IgG1 antibody against the p19 subunit of IL-23, did not lead to significant improvement [37]. The reason why IL-23 inhibitors do not significantly improve RA is unclear. However, the IL-23/IL-17 axis may be associated with not only acquired immunity, but also innate immunity because innate immune cells like γδ T cells or mast cells produce IL-17 [38]. The effect of IL-23 inhibitors on inflammatory diseases may depend on the contribution of the innate immune system. 

In addition to IL-23 inhibitors, IL-17 inhibitors such as secukinumab also do not significantly improve RA. The reason why IL-23/IL-17 targeted therapy for RA failed is not clearly understood, but there are two possibilities. First, as described above, the IL-23/IL-17 axis may contribute to the pathogenesis of innate immunity rather than acquired immunity. Second, the IL-23/IL-17 axis may affect the clinical course of RA patients only at the onset of arthritis. 

### 2.2. IL-23 and Spondylarthritis (AS and PsA)

The IL-23/Th17 pathway is associated with spondylarthritis (SpA) including ankylosing spondylitis (AS) and psoriatic arthritis (PsA) [39,40]. It was reported that susceptibility to AS is associated with polymorphisms at four genetic loci including the IL-23 receptor *(IL23R), ERAP1*, *IL1R2*, and *ANTXR2*, which established a major role for IL-23 in the pathogenesis of AS [41]. Furthermore, polymorphisms in STAT3 are associated with AS [42]. In addition, IL-17 and IL-23 concentrations in serum increased in AS patients [43]. In facet joints, which are the characterized lesion in AS, IL-17-producing cells also increased [44]. In an animal model of arthritis, Sherlock et al. reported that IL-23 acts on entheseal resident T cells expressing the IL-23 receptor and ROR-γt, and that IL-23 induces inflammatory cytokines including IL-17 and IL-22 [45]. They also showed that IL-22 stimulated with IL-23 induces new bone formation by osteoblasts at tendon-bone attachments, which suggests that IL-23 dysregulation results in both enthesitis and entheseal new bone formation even in the absence of synovitis. 

The IL-23/IL-17 axis is also involved in the pathogenicity of PsA [46]. In addition to AS, polymorphisms in *IL23R* are a risk factor for PsA [47]. The frequency of Th17 cells is increased in PsA synovial fluid [48]. Celis et al. found an association between higher IL-23A mRNA expression and synovial lymphoid angiogenesis, and that IL-23A mRNA expression significantly correlates with swollen joint count and CRP in PsA patients [49]. In addition, Raychaudhuri et al. showed that IL-17 induces MMP-3 and proinflammatory cytokines such as IL-6 from cultured FLS with PsA patients [48]. Furthermore, IL-22/IL-22R could be a therapeutic target for the treatment of PsA [50]. 

There are no reports which clearly explains the differences of pathogenicity between PsA and AS. As described above, bone formation followed by bone resorption in the characteristic manifestation SpA including PsA and AS. Dickkopf-1 (Dkk-1), a Wnt signal inhibitor, is a bone turnover marker and elevated Dkk-1 level reflects inhibition of osteoblasts differentiation. Serum concentrations of Dkk-1 are elevated in PsA patients with “peripheral” arthritis [51]. In contrast, serum concentrations of Dkk-1 are decreased in axial spondyloarthritis patients, reflecting the highly progression of newly bone formation at the spine [52]. Thus, bone turnover could be different from PsA patienets with only peripheral arthritis and AS patients. However, the differences of pathogenicity and therapeutic effect between PsA and AS remains unclear. Therefore, further investigation is needed to clarify the precise mechanism in each inflammatory arthritis. 

For the treatment of AS, TNF inhibitors including infliximab, adalimumab, golimumab, and etanercept are known to improve clinical manifestations [53]. Recently, new biological agents targeting the IL-23/IL-17 axis have been developed. Secukinumab, a fully human monoclonal IgG1κ antibody against IL-17, was reported to decrease clinical symptoms in AS patients [54]. Furthermore, ustekinumab, a fully human monoclonal IgG1κ antibody against the p40 subunit of IL-12 and IL-23, improved the Bath Ankylosing Spondylitis Disease Activity Index (BASDAI) score [55]. 

Secukinumab also significantly reduces clinical symptoms and inhibits radiographic progression [56]. Furthermore, ixekizumab, humanized IgG4 against IL-17, significantly improves disease activity and physical function by inhibiting bone destruction in biologic-naïve patients with active PsA [57]. The IL-23 inhibitor ustekinumab decreases clinical manifestations of peripheral arthritis, dactylitis, enthesitis, as well as psoriasis [58]. Furthermore, guselkumab, a fully human IgG1 antibody against the p19 subunit of IL-23, is highly-effective in psoriasis patients [59]. Guselkumab may be a useful agent against psoriatic arthritis in the future. 

## 3. Conclusions

**Figure 1:**
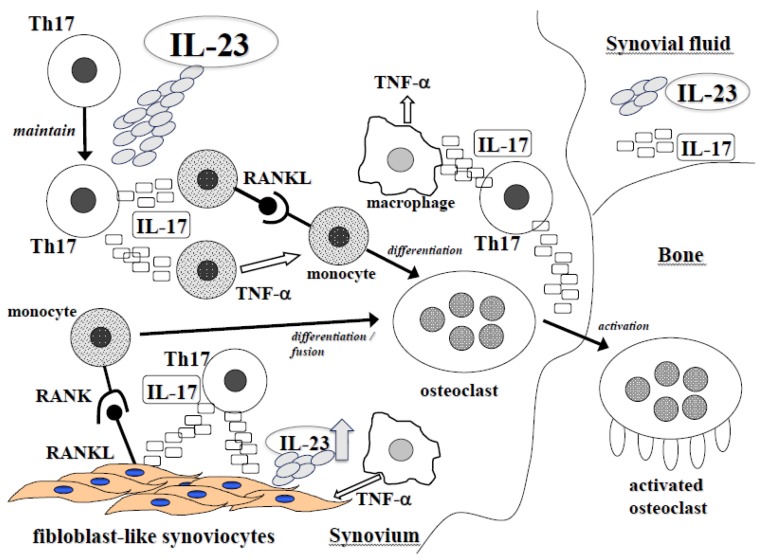
The role of IL-23 in inflammatory arthritis including RA and SpA. IL-23 maintains T17 cells and stimulates IL-17 production. IL-17 induced by IL-23 increases the production of inflammatory mediators such as TNF-α. Furthermore, IL-17 induced by IL-23 up-regulates the expression of RANKL. TNF-α and RANKL cooperates osteoclastogenesis synergistically. The expression of IL-23 is up-regulated by IL-17 and TNF-α.

**Figure 2:**
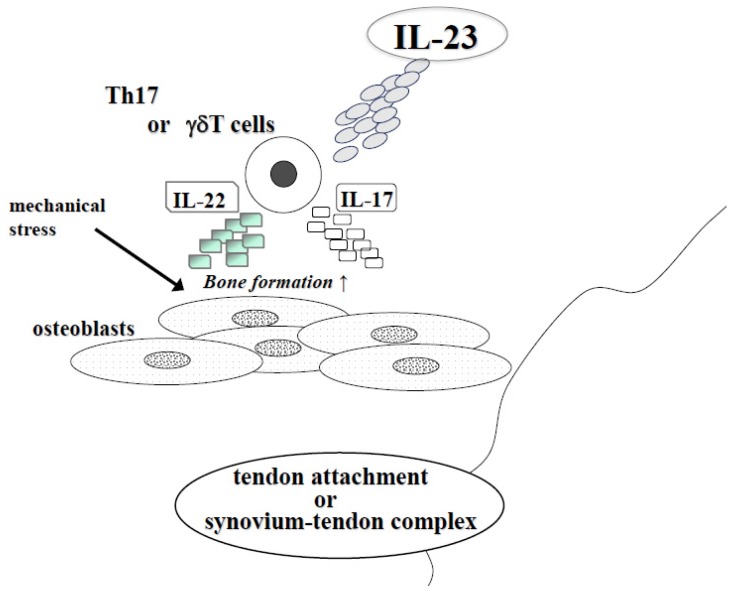
The effect of IL-23 in SpA at tendon attachment or synovium-tendon complex. In SpA, new bone formation develops at tendon attachment or synovium-tendon complex. IL-23 induced IL-17 or IL-22 from Th17 or γδT cells and especially IL-22 stimulates osteoblast differentiation. At these sites, mechanical stress also stimulated bone formation.

The IL-23/IL-17 axis is involved in the pathogenesis of RA and SpA (Figure 1 and Figure 2). The effects of IL-23 can be explained by both inflammation and bone destruction. IL-23 is a useful marker for the diagnosis of RA and anti-IL-17 and anti-IL-23 antibodies have great efficacy for AS and PsA. Understanding of IL-23 and Th17 cells may help control the disease progression of autoimmune arthritis.
